# Working in the Woodlands: A mixed methods evaluation of Green Care in First Episode Psychosis

**DOI:** 10.1192/j.eurpsy.2022.532

**Published:** 2022-09-01

**Authors:** H. Sharp, C. Berry, S. Cuthbert

**Affiliations:** 1 Brighton and Sussex Medical School, Psychiatry, Brighton, United Kingdom; 2 Sussex Partnership NHS Foundation Trust, Psychiatry, Worthing, United Kingdom

**Keywords:** Nature-based care, Green Care, New intervention, First Episode Psychosis

## Abstract

**Introduction:**

Recognition of the essential role of nature-based activities for general wellbeing is expanding. Currently, there is limited evidence of the benefits of green care for those with severe and enduring mental illness, including psychosis.

**Objectives:**

We aim to establish benefits and difficulties encountered during a 10-session green care programme for 18-30 year olds who have experienced first episode of psychosis (FEP) using a mixed methods approach.

**Methods:**

This was a service evaluation of a ’Woodland Group’ of 10 half-day sessions for participants with FEP. Sessions consisted of a welcome and agenda setting, ice-breaking activity, core nature-based activity. Quantitative data for this evaluation was collected through the 15-item Questionnaire on the Process of Recovery (QPR), and a semi-structured intervention experience questionnaire. Qualitative data was collected via a focus group. Thematic analysis was performed by the three co-authors.

**Results:**

4/8 patients showed reliable improvement on QPR outcome measures, 1 showed deterioration and 3 showed no change. Mean QPR scores showed modest increase from average 3.4 (week 1) to 3.8 (week 10). 100% of respondents would recommend this group to others. Thematic analysis identified themes of connection with nature and others, development of a sense of wellbeing and ‘peacefulness’ and new perspectives on psychotic experience.

**Conclusions:**

This small, retrospective evaluation is the first to investigate green care interventions for young people experiencing FEP. Our results reflect the positive informal feedback from participants and supporting staff. Limitations include small sample size, incomplete data, and reliance on patient-reported outcomes. These findings show promise for nature-based activities within EIS.

**Disclosure:**

No significant relationships.

